# Integrated Numerical Simulations and Experimental Measurements for the Sintering Process of Injection-Molded Ti-6Al-4V Alloy

**DOI:** 10.3390/ma15228109

**Published:** 2022-11-16

**Authors:** Shaohua Su, Zijian Hong, Yuhui Huang, Peng Wang, Xiaobao Li, Junwen Wu, Yongjun Wu

**Affiliations:** 1 School of Materials Science & Engineering, Zhejiang University, Hangzhou 310027, China; 2 Jiangsu Gian Technology Co., Ltd., Changzhou 213016, China; 3 Cyrus Tang Center for Sensor Materials and Applications, State Key Laboratory of Silicon Materials, Zhejiang University, Hangzhou 310027, China

**Keywords:** Ti-6Al-4V alloy, metal injection molding, numerical simulation, sintering process

## Abstract

Metal injection molding (MIM) is an advanced manufacturing technology that enables the mass production of high-performance and complex materials, such as the Ti-6Al-4V alloy. The determination of the size change and deformation of the Ti-6Al-4V alloy after the sintering process is challenging and critical for quality control. The numerical simulation could be a fast and cost-effective way to predict size change and deformation, given the large degrees of freedom for the sintering process. Herein, a finite element method based on the thermal-elastic-viscoplastic macroscopic model is developed to predict the shrinkage, deformation, relative density, and crack of injection-molded Ti-6Al-4V after sintering, using the Simufact software. Excellent agreements between experimental measurements and numerical simulations of the size and deformation are demonstrated (within a 3% error), confirming the accuracy of the numerical model. This approach can serve as a guideline for the mold design and sintering optimization of the MIM process.

## 1. Introduction

The Ti-6Al-4V alloy has advantages, including its low density, high strength, good biocompatibility, and excellent heat and corrosion resistance, as well as being non-magnetic and luxurious [1]. Such superior features promote its applications in various fields, such as aerospace, transportation, chemical, medical, and wearable industries [2,3,4]. Due to the relatively low thermal conductivity (about 1/5 of iron) and Young’s modulus (about half of iron) [5,6,7], the Ti-6Al-4V alloy is difficult to be processed by traditional methods, such as machining or forging. As one of the state-of-the-art advanced manufacturing technologies, metal injection molding (MIM) has become the major manufacturing technique for complex Ti-6Al-4V materials, with a relatively low-cost and near-net-shape process [8,9,10,11,12,13,14]. MIM combines the shape-making complexity of plastic injection molding with the material flexibility of powder metallurgy, which is composed of four sequential steps: mixing, injection molding, debinding, and sintering [15,16].

Sintering is one of the key processes for MIM that determines the accuracy and performance of the materials. It is a thermal process below the melting point of the main constituent, which could facilitate particle bonding through solid-state diffusion that increases structural strength and density and reduces system energy [17,18,19,20]. The sintering phenomenon involves particle fusion, volume reduction, porosity reduction, and grain growth. This process is largely influenced by multiple factors, including particle size, particle shape, composition, initial density, heating rate, sintering peak temperature, sintering holding time, sintering pressure, sintering atmosphere, etc. [21,22,23]. For instance, the brown part before sintering is a porous packing of loose powder held together by weak surface bonds with initial porosity of ~35% to 50%. During sintering, the individual particles fuse to create a dense, strong monolithic part, which undergoes a large shrinkage during the sintering process, generally 10% to 20% [24].

Because of the large shrinkage in the sintering process, determination of the size change and deformation is necessary yet challenging for industrial mass production to produce the materials in near-net-shape and high quality. Given the complexity of the parameter space for the sintering process, as compared to the conventional trial and error method, numerical simulation of the sintering stage could be a fast and cost-effective alternative to predict the size change and deformation [25]. This could enable the accurate design of the furnace and sintering parameters (sintering temperature, heating rate, etc.) towards customized products with controlled quality while largely minimizing the experimental time and cost. Currently, there are different methodologies to model sintering processes, including continuum, micromechanical, multiparticle, and molecular dynamics approaches, which cover multiple length scales. Among these methodologies, continuum models, such as finite element analysis, could predict relevant attributes, such as shrinkage, deformation, and density profile, at a relatively large length scale [26,27,28,29,30]. Nosewicz et al. [31] presented a viscoelastic model of powder sintering developed within the discrete element framework. This model has been applied to simulate the free and pressure sintering process of Ni-Al. Mohsin et al. [18] used a finite element (FE) method based on a thermo-kinetic model to describe the densification process of MIM copper during sintering. The research focused on measuring thermos physical properties and numerical simulation associated with the sintering process. Furthermore, some enhancements were suggested in the temperature field calculation of the FE model to mimic a real furnace condition. Jeong et al. [32] developed a unified model for describing compaction and sintering based on plasticity theory. A method for predicting the final dimensions of a powder material was proposed. The proposed model simulated the powder process continuously and simultaneously and was more effective than previous models that treat compaction and sintering separately. Kwon et al. [16] studied the simulation of the sintering densification and shrinkage behavior of powder-injection-molded 17-4 PH stainless steel. The predictive capability of the model was verified by comparing the theoretical calculations with the experimentally observed variation in sintering shrinkage of the samples determined by dilatometry. Sahli et al. [21] studied the sintering of micro-parts using the powder injection molding process, with numerical simulations and experimental analysis. They concluded that the FE simulation results had better agreement with the experiments at high temperatures. Although the finite-element-based continuum simulation method has been widely employed in the modeling of the sintering process, there are barely studies on the numerical simulation and experimental analysis of the Ti-6Al-4V sintering process by MIM. As compared to other metal materials, Ti-6Al-4V by MIM has lower maturity, higher experimental cost, and greater difficulty in densification and dimensional control; the integrated theoretical and experimental understandings for the sintering process of injection-molded Ti-6Al-4V is, thus, greatly needed.

Herein, we aim to simulate the shrinkage, deformation, relative density, and crack of the injection-molded Ti-6Al-4V after sintering, using the finite element method. Thermo-elasto-viscoplastic constitutive law based on continuum mechanics was employed to describe the sintering process. Numerical simulations of the Ti-6Al-4V round and elongated specimens were carried out with the Simufact software and compared with experimental results. It is shown that our finite-element-based sintering model could accurately predict the densification behaviors of the specimens (within a 3% error, as compared to the experimental data). This study can serve as a guideline and example for the mold design and sintering optimization of the MIM process towards industrial applications.

## 2. Methods

**Figure 1:**
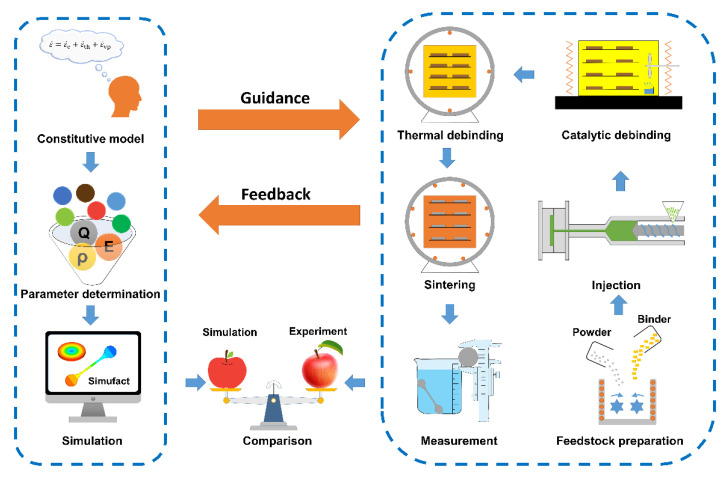
Schematic diagram of the workflow in this study.

The working flow of this research is shown in Figure 1. Numerical simulation and experiment of Ti-6Al-4V sintering by MIM were carried out in parallel to do a point-by-point comparison. For numerical simulation, based on the continuum mechanics theory, a macroscopic sintering model based on the viscoplastic constitutive law (details given in Section 3) was established first, and then the parameters for Ti-6Al-4V were determined. Finally, the numerical simulations for the Ti-6Al-4V round specimen and elongated specimen were carried out. The FE method is a numerical computational method for solving a system of differential equations through approximation functions applied to each element, called domain-wise approximation. This method is very powerful for the typical complex geometries encountered in powder metallurgy. The factors affecting the sintering shrinkage and deformation of MIM parts, such as gravity, friction, and temperature, were analyzed [26]. Simufact software with a sintering module was used for the FE simulation to avoid unnecessary cost and time expenditures and improve materials quality [33].

**Figure 2:**
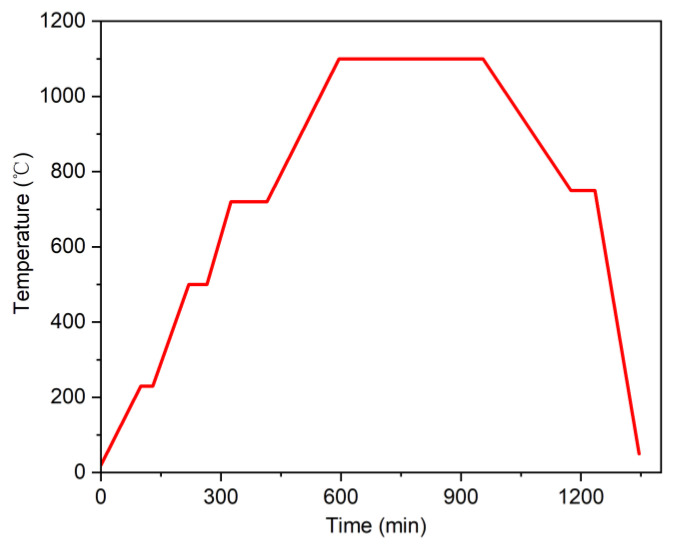
A typical sintering curve for Ti-6Al-4V.

On the other hand, Ti-6Al-4V was sintered experimentally using MIM. High pure atomized Ti-6Al-4V powder (Avimetal PM, Beijing, China) with a spherical shape was used as the raw material in this study. The feedstock was prepared with a polyformaldehyde (POM) -based binder system. The green parts were injected into an injection molding machine (NEX80IIIT, NISSEI, Nagano-ken, Japan) at a maximum injection pressure of 130 MPa. The injection temperatures from barrel to nozzle were 50 °C, 170 °C, 180 °C, 185 °C, 190 °C, and 195 °C. Catalytic debinding was performed in a catalytic debinding furnace (STZ-400L-OA, Sinterzone Company, Shenzhen, China) at 130 °C for 7 h with nitric acid as the catalytic medium. This process was mainly used to remove POM. Thermal debinding was performed in a graphite furnace (VM40/40/150, Hiper, Ningbo, China) at 700 °C for 2.5 h to remove the backbone binder. The sintering process was performed at 1100 °C for 6 h in a vacuum sintering furnace (pressure < 10^−3^ Pa, VM42/45/125, Hiper, Ningbo, China). The sintering curve of Ti-6Al-4V is shown in Figure 2. The density of product specimens was measured by the Archimedes method with deionized water. The relative density was calculated as the ratio of the measured density to the theoretical density. Dimension was measured by vernier caliper.

Eventually, the numerical simulation and experiment results were compared apples to apples to verify the correctness of the sintering model.

## 3. Constitutive Model of Sintering Process

### 3.1. Principle

The sintering process provides energy to bond the individual powder particles together to eliminate the pores between particles. The sintering process can be divided into three stages: (1) aggregation of the particles and disappearance of the borders begin to produce a neck in the points of contact between particles. Grain boundaries are formed between two adjacent particles in the contact plane. The centers of particles are only slightly closer with very low shrinkage. (2) Pores are reduced due to the growth of the neck. These pores are reorganized and interconnected like a cylindrical channel to maximize the contact between the particles. The grain growth takes place later in this stage. (3) Pores are closed, isolated, and located principally at the boundaries or within the grains. This step is much slower than the first two stages. Additionally, the densification rate becomes slow and the grain growth is more evident [22].

The brown part composed of powders and pores after debinding is considered as a compressible continuum media in the macroscopic model. This porous particle has high surface energy, and, as the temperature increases, the movement of atoms intensifies, which decreases the surface energy. A viscoplastic constitutive law is adopted to describe the densification behavior of powders. In addition, the effects of elasticity and thermal expansion are taken into account. The densification process of sintered materials is affected by the evolution of the temperature field [34]. During the sintering process, elastic strain, thermal strain, and elastic–plastic strain are affected by the temperature change. Diffusion process, grain growth, creep, surface tension effect, gravity, friction, and thermal dependence are considered in the sintering process, with the coupling of several thermo-mechanical phenomena.

### 3.2. Thermo-Elasto-Viscoplastic Constitutive Law

Thermo-elasto-viscoplastic constitutive law is employed to describe the sintering process. The total strain rate $\dot{\varepsilon }$ consists of three parts: elastic strain rate ${\dot{\varepsilon }}_{e}$, thermal strain rate ${\dot{\varepsilon }}_{th}$, and viscoplastic strain rate ${\dot{\varepsilon }}_{vp}$, i.e., [35]

(1) $$\dot{\varepsilon }={\dot{\varepsilon }}_{e}+{\dot{\varepsilon }}_{th}+{\dot{\varepsilon }}_{vp}$$

The elastic strain rate and thermal strain rate are due to the change in furnace temperature [36]. The elastic strain rate is assumed to be linear and isotropic and can be expressed with Hooke’s law [22]:

(2) $$\dot{\sigma }=k_{e}{\dot{\varepsilon }}_{e}$$

where $\dot{\sigma }$ is the stress rate and $k_{e}$ is the elastic stiffness matrix while the thermal strain rate caused by thermal expansion can be expressed as [21]:

(3) $${\dot{\varepsilon }}_{th}=\alpha \Delta \dot{T}I$$

where α is the material thermal expansion coefficient, $\Delta \dot{T}$ is the temperature change rate, and I is the second order identity tensor.

When the temperature exceeds a certain transition temperature, viscoplastic strain instead of elastic strain dominates the sintering process, and the specimen begins to densify. Viscoplastic strain refers to the creep of materials at high temperature due to grain boundary diffusion and lattice diffusion [17]. According to the linear viscoplastic theory, the viscoplastic strain rate can be expressed as [34]:

(4) $${\dot{\varepsilon }}_{vp}=\frac{\sigma^{\prime }}{2G_{p}}+\frac{tr\left(\sigma \right)-3\sigma_{s}}{9K_{p}}I$$

where σ is the stress tensor in sintered materials, $tr\left(\sigma \right)$ is the trace of the stress tensor, $\sigma^{\prime }$ is the deviatoric stress tensor, $\sigma_{s}$ is the sintering stress, and $G_{p}$ and $K_{p}$ are the shear viscosity modulus and bulk viscosity modulus of the material, respectively. The first term on the right side of the equation determines deformation during sintering, and the second term that is originated from the hydrostatic stress determines the volume shrinkage during sintering [26].

Sintering stress, which changes with the evolution of microstructure during different sintering stages, is the driving force for densification due to interfacial energy reduction [16,24]. Initially, the sintering stress is related to the particle size and sintering neck size in the early stage. Then, the pores in the sintered part are interconnected and cylindrical, so sintering stress is related to the curvature of the pores. Finally, the grains grow up rapidly, and the pores are located at the intersection of grain boundaries. Sintering stress depends on the size of grains and pores. Since shrinkage mainly occurs in the middle stage of the sintering process where the grain growth is not obvious, the following equation can be used to approximate the sintering stress [35]:

(5) $$\sigma_{s}=\frac{C\rho^{2}}{r_{p}}$$

where $r_{p}$ is the radius of powder, ρ is the density of the sintered material, and C is a material parameter, which is a function of surface free energy.

Both shear viscosity modulus and bulk viscosity modulus depend on relative density, temperature, and microstructural factors, such as grain and pore size [16,22]. The shear and bulk viscosity modulus for the sintered materials are defined as the following [21,24]:

(6) $$G_{p}=\frac{\eta_{p}}{2\left(1+\upsilon_{p}\right)}$$

(7) $$K_{p}=\frac{\eta_{p}}{3\left(1-2\upsilon_{p}\right)}$$

where $\eta_{p}$ and $\upsilon_{p}$ are the uniaxial viscosity and viscous Poisson’s ratio of the material, respectively [17].

### 3.3. Steady Creep Law

Creep is considered the major source for the viscoplastic behavior [37]. Steady-state creep rate, which is affected by material properties and environment, is the most important index to evaluate the creep resistance of materials at high temperatures. It is generally expressed in terms of an Arrhenius-like relation [38]:

(8) $${\dot{\varepsilon }}_{s}=A\sigma^{n}exp\left(-\frac{Q}{RT}\right)$$

where ${\dot{\varepsilon }}_{s}$ is the steady-state strain rate (s^−1^), A is the pre-exponential constant, Q is the creep activation energy, R is the gas constant, σ is the applied stress (in Pa), n is the stress exponent, and T is the temperature.

### 3.4. Relative Density

In a sintering process, particles connect and fuse together while the pores among the particles reduce gradually. Macroscopically, the sintered parts shrink with the volume reduction and increase in the density. The change of relative density during sintering conforms to the mass conservation law, where the mass of the powder conserves with the gradual disappearance of the pores in the brown parts [34]:

(9) $$\dot{\rho }=-\rho tr\left(\dot{\varepsilon }\right)$$

where $tr\left(\dot{\varepsilon }\right)$ is the trace of total strain rate.

### 3.5. Friction

Friction acts as an obstacle to shrinkage, and the greater the friction, the less sufficient the shrinkage. The friction (f) can be expressed as:

(10) f=μN

where μ is the sliding friction coefficient, which is related to the material and contact surface roughness; N is the positive pressure. If N is in the vertical direction, it equals the gravity.

## 4. Simulation

Commercially available software Simufact (https://www.simufact.com/, accessed on 20 September 2022) was used for numerical simulations in this study, based on the above models.

### 4.1. Parameter

To simulate the process, it is necessary to specify the basic properties of the material, which are temperature-dependent [22,33]. Most of the material parameters for Ti-6Al-4V are already predefined by the software vendor. The properties for Ti-6Al-4V used in the simulation are tabulated in Table 1. Powder distribution is considered homogeneous.

**Figure 3:**
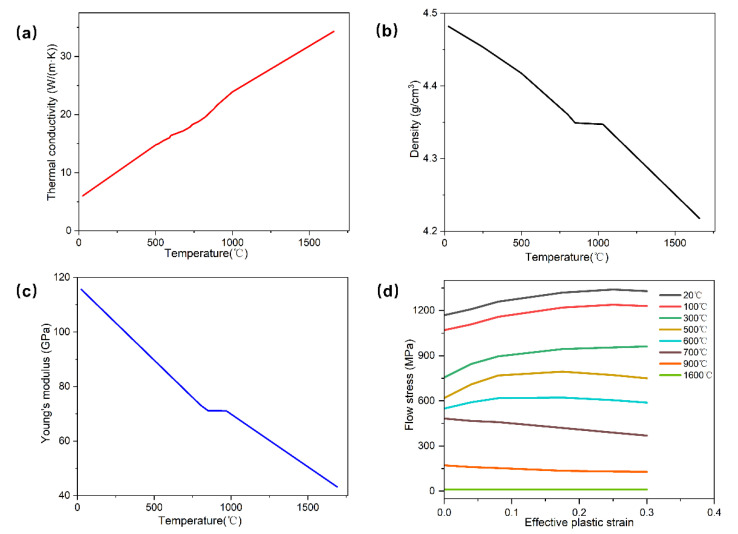
Physical properties of Ti-6Al-4V used in the simulations. (**a**) Thermal conductivity, (**b**) density, (**c**) Young’s modulus, and (**d**) flow curves.

Figure 3 shows the additional physical properties of Ti-6Al-4V used for the simulations. As shown in Figure 3a, as the temperature increases from room temperature, the thermal conductivity increases almost monotonically from 5 W/(m·K) to 35 W/(m·K), which is consistent with the previous report [39], while due to the thermal expansion, the density of Ti-6Al-4V decreases slightly with increasing temperature (Figure 3b). Notably, the density plateau around 985 °C can be attributed to the α + β ↔ β phase transformation. Whereas the Young’s modulus gradually decreases with the increase in temperature, owing to the softening of the material at elevated temperatures, as shown in Figure 3c. The mechanical response of Ti-6Al-4V is expressed through flow stress curves at different temperatures (from 20 °C to 1600 °C), which is presented in Figure 3d. The material is easier to deform with the decrease in flow stress at higher temperatures.

### 4.2. Round Specimen Simulation

**Figure 4:**
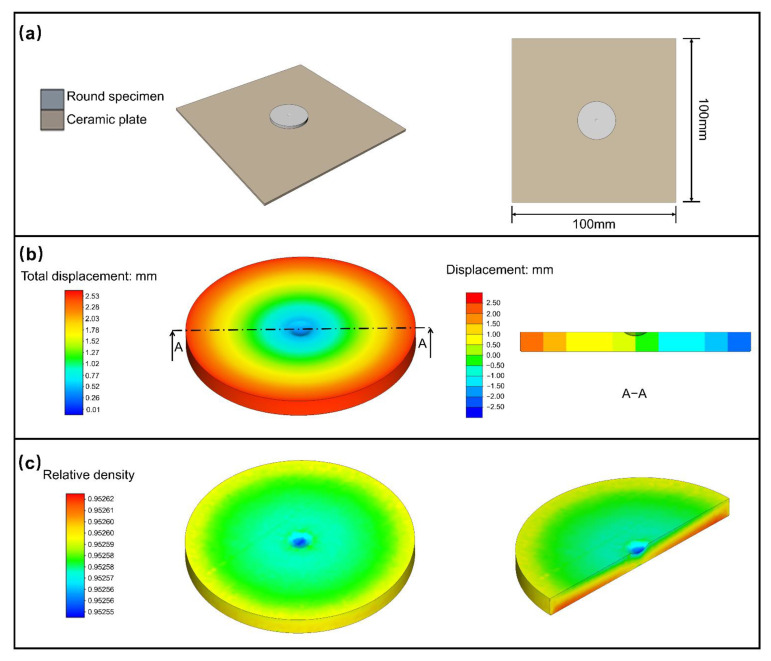
Simulation of round specimen for Ti-6Al-4V. (**a**) Layout, (**b**) total displacement, and (**c**) relative density.

As shown in Figure 4, a round specimen is selected for the first simulation. The numerical simulation aims to predict the density variations and shrinkages of the 3D specimens in sintering. The dimension of the ceramic plate is set as 100 mm × 100 mm × 2 mm (Figure 4a), and the round specimen is placed on top of a ceramic plate. It can be observed that after sintering, the round specimen shows a negative/positive displacement on the center/edge (Figure 4b), indicating a central symmetrical shrinking of the sample from the center of the sample. This can be understood given that the center of the round specimen is the gate for injection. As shown in Figure 4c, the relative density of the specimen is about 95.26%, and the sintering process makes the final density of sintered specimen almost uniform. The uniformity of sintered density is also related to factors, such as green density distribution, specimen shape, and sintering temperature field. A slightly lower density can be seen in the center of the round specimen that could be due to the pull effect from surrounding positions during sintering.

The parameter comparison of the round specimen for Ti-6Al-4V is presented in Table 2. The errors are very small for dimension and density, which means the simulation values are in good agreement with the experimental measurements. Since the density simulation comprehensively considers the shrinkage in different directions, the density error is relatively larger than that of the dimension. Due to the regular shape of the round specimen, it exhibits linear shrinkage during sintering.

### 4.3. Elongated Specimen Simulation

**Figure 5:**
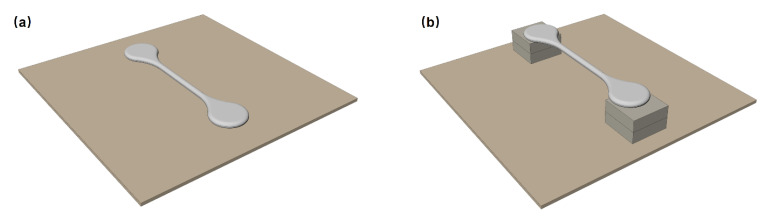
Elongated specimen of Ti-6Al-4V. (**a**) Laid and (**b**) supported.

As shown in Figure 5, elongated specimens were carried out for the second simulation. There are two sintering modes, one is laid flat on the ceramic plate (Figure 5a) and the other is supported by ceramic blocks on both ends (Figure 5b). The overall length, width, and thickness of the green specimen are 114 mm, 23 mm, and 3.8 mm, respectively. For the second sintering mode, the span between ceramic blocks is 80.7 mm.

**Figure 6:**
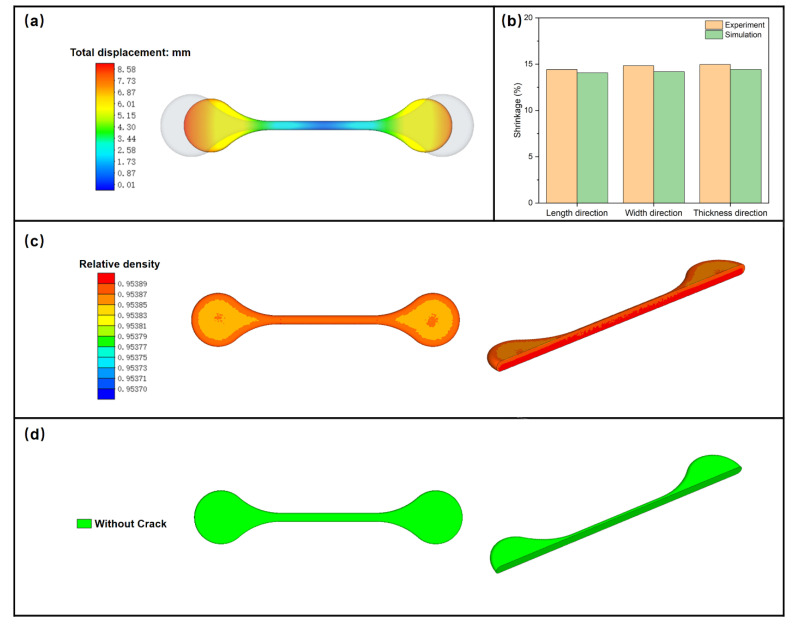
Properties of the elongated specimen for laid Ti-6Al-4V. (**a**) Total shrinkage, (**b**) shrinkage along different directions, (**c**) relative density, and (**d**) crack.

Properties of the elongated specimen for the laid Ti-6Al-4V are shown in Figure 6. The gray and colored contours in Figure 6a represent the specimen before and after sintering, respectively. During thermal debinding and sintering, binder elimination and subsequent particles bonding take place, resulting in the dimensional change of the MIM parts. Compared with the green part, the dimensional change after debinding was not noticeable whilst the dimensional change after sintering was clearly evident [36,40]. The shape of the elongated specimen is more complex than that of the round specimen, which has multiple shrinkage centers during sintering, resulting in a higher nonlinearity of shrinkage. The variations in particle size, composition, mold wear, furnace temperature distribution, and other factors, such as reactions between particles during sintering, all affect the dimensional control of the MIM parts [26]. It can be seen that the simulated and experimental values have good consistency in length, width, and thickness direction (Figure 6b), which shows the accuracy of the numerical simulation. The final density is depicted in Figure 6c, which shows an almost homogenous distribution with a value of ~95.4%. In some cases, cracks can be formed in the sintered specimen due to the high thermal or stress gradients. In this study, no crack was found in the simulations (Figure 6d) or experiments, which shows that the rationality of the sintering curve had well-designed heating and cooling rates.

**Figure 7:**
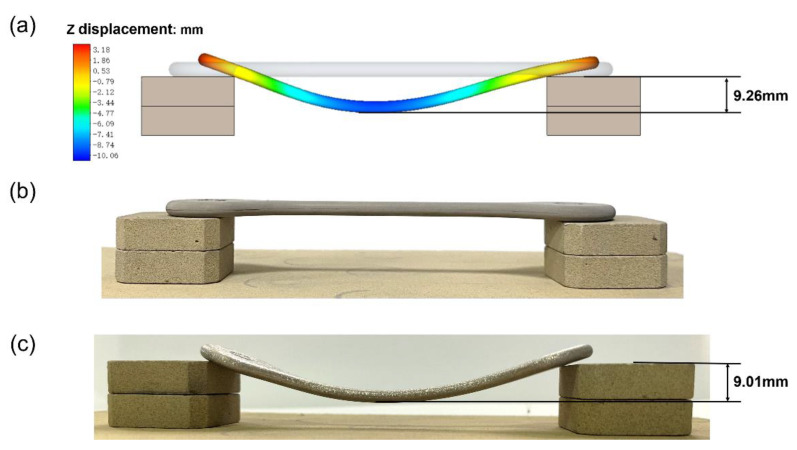
Deformation of elongated specimen for supported Ti-6Al-4V. (**a**) Numerical simulation, (**b**) specimen after thermal debinding, and (**c**) specimen after sintering.

The deformation of the elongated specimen for the supported Ti-6Al-4V from the simulation and experiment is also shown in Figure 7. The gray and colored contours in Figure 7a represent the specimen after thermal debinding and sintering from simulation, respectively. On the other hand, Figure 7b,c show the experimental results of the specimen after thermal debinding and sintering, respectively. It is discovered that the specimen has almost no deformation and shrinkage after thermal debinding. For specimens after sintering, the farther away from the support block, the greater the deformation, which is due to the effect of the gravity. A maximum deformation of 9.26 mm can be observed after sintering, consistent with the experimentally measured value of 9.01 mm, giving rise to a relative error of only ~2.8%, showing the accuracy of the numerical model. In the sintering process, the specimen bended due to its own gravity. According to Equation (4), shrinkage in the sintering process depends on the ratio of sintering stress and bulk viscosity modulus, while the sintering deformation depends on the shear viscosity modulus. Shrinkage can be simulated with greater accuracy than that of deformation.

MIM specimens reach very low strength levels during sintering. Accordingly, weak forces, such as gravity, friction, and non-uniform heating, induce deformation [26]. Since gravity only acts on the vertical direction, the specimen shrank unevenly and deformed in the sintering process. The effect of gravity on the shrinkage and deformation of sintered specimens is related to the size of the specimen and the type of ceramic block used in sintering. At the same time, the friction between porous specimens and ceramic blocks also led to the sintering deformation. The deformation of the specimen results in the change of stress distribution in which the compressive stress is beneficial to the densification of sintering, while the tensile stress is contrary.

The experimental relative density is very close to the simulated value, whether laid or supported. Interestingly, the experimental values of sintered density are slightly larger than the simulated ones, which is understandable given that the simulation is carried out under ideal vacuum conditions, where only the role of thermal radiation is considered. Although there is still appropriate heat conduction and convection, in addition to thermal radiation experimentally, the specimen is subjected to more heat action with an additional heat source.

## 5. Conclusions

In conclusion, we have developed an FE method based on the thermal-elastic-viscoplastic macroscopic model to predict the shrinkage, deformation, relative density, and crack of injection-molded Ti-6Al-4V after sintering, using the commercially available Simufact software. Experiments were simultaneously carried out to justify the accuracy of the sintering model and simulation method. The results showed a good agreement (within a 3% error) between the experimental measurements and numerical simulations. The slightly larger sintered density and shrinkage in the experiment than those in the simulation are due to additional thermal convection and conduction during the sintering experiments. The deformation was affected by gravity, friction, specimen shape, and support mode. This approach can serve as a guideline for mold design and sintering optimization and could be extended to other MIM materials while a follow-up study on the influence of sintering temperatures and material’s compositions is needed to further extend this method.

## Figures and Tables

**Table 1:** Materials parameters for Ti-6Al-4V used in the simulation.

Menu	Parameter	Value	Unit
General properties	Gravitational acceleration	9.8	m/s^2^
	Initial relative density	60.1	%
	Friction coefficient	0.3	-
	Pre-exponential constant	2.36 × 10^−36^	1/(Pa·s)
	Creep activation energy	277	kJ/mol
	Gas constant	8.31	J/(mol·K)
Thermal properties	Specific heat capacity	540	J/(kg·K)
	Melting temperature	1640	°C
	Latent heat for melting	419	J/g
	Thermal expansion coefficient	8.84 × 10^−6^	1/K
Mechanical properties	Ultimate strain	12	%
	Stress exponent	5.64	-
	Poisson’s ratio	0.34	-
	Yield strength	941	MPa
	Tensile strength	1000	MPa

**Table 2:** Parameters comparison for round specimen of Ti-6Al-4V.

Parameter	Before Sintering	Experiment after Sintering	Simulation after Sintering	Error
Diameter (mm)	34.80	29.78	29.90	0.4%
Thickness (mm)	2.91	2.48	2.49	0.4%
Relative density (%)	60.10	97.80	95.26	−2.6%

## Data Availability

The data are available from the corresponding author (hongzijian100@zju.edu.cn) upon reasonable request.
